# Recent Advances in Flexible Pressure Sensors: Mechanisms, Materials, Designs and Applications

**DOI:** 10.3390/s26154993

**Published:** 2026-08-06

**Authors:** Xiuzhen Yang, Chaojin Chen, Meng Wang, Kai Yao, Jiaoyue Zhang, Jiayi Lu, Ying Yi

**Affiliations:** School of Mechanical Engineering and Electronic Information, China University of Geosciences, Wuhan 430074, China; yangxiuzhen@cug.edu.cn (X.Y.); chenchaojin@cug.edu.cn (C.C.); wm0302@cug.edu.cn (M.W.); yaokai0625@cug.edu.cn (K.Y.); zhangjiaoyue@cug.edu.cn (J.Z.); lujiayi@cug.edu.cn (J.L.)

**Keywords:** flexible pressure sensors, wearable electronics, smart healthcare, human–machine interaction, motion monitoring

## Abstract

In recent years, the rapid development of flexible electronics, smart materials, and micro/nanofabrication technologies has greatly promoted the advancement of flexible pressure sensors. These sensors have achieved significant improvements in sensitivity, detection range, stability, and functional integration, demonstrating great potential for applications in wearable electronics, smart healthcare, and human–machine interaction. This review summarizes recent progress in flexible pressure sensors in terms of sensing mechanisms, functional materials, structural designs, and intelligent applications. First, the working principles and performance characteristics of typical sensing mechanisms, including piezoresistive, capacitive, piezoelectric, triboelectric, iontronic, self-powered, and electrochemical sensing, are introduced and compared. Then, the development of key materials, such as flexible substrates, carbon-based nanomaterials, metal nanostructures, conductive hydrogels, and MXenes, is summarized. The effects of structural designs, including serpentine, three-dimensional porous, crack, wrinkle, and Kirigami structures, on flexibility, stretchability, sensitivity, detection range, and cycling stability are also discussed. Furthermore, the applications of flexible pressure sensors in pulse monitoring, blood pressure monitoring, human motion detection, cardiovascular health assessment, disease diagnosis, gesture recognition, human–machine interaction, and electronic skin are reviewed. Finally, the major challenges and future perspectives of flexible pressure sensors are discussed.

## 1. Introduction

Sensors are essential components of modern information systems. They play a key role in artificial intelligence (AI), industrial automation, the Internet of Things (IoT), and mechatronics. Traditional rigid sensors, especially silicon-based sensors, have made significant progress in miniaturization and high-sensitivity detection. However, these sensors have several inherent limitations. They are mechanically brittle, relatively dense, and difficult to conform closely to complex curved surfaces. These limitations restrict their use in emerging fields such as wearable electronics, smart healthcare, and flexible human–machine interaction.

In recent years, flexible electronics, advanced functional materials, and micro- and nanofabrication technologies have developed rapidly. These advances have promoted the development of flexible sensors. Compared with conventional rigid sensors, flexible sensors offer excellent mechanical flexibility, large deformation capability, good biocompatibility, and low power consumption. Therefore, they have become an important research direction in the field of intelligent sensing [1,2,3].

Among various types of flexible sensors, flexible pressure sensors can convert pressure stimuli into electrical signals. They have broad application potential in health monitoring, human–machine interaction, and electronic skin. However, several key challenges remain as these devices move toward intelligent and multifunctional systems. For example, conventional flexible piezoresistive pressure sensors often have limited stretchability and pronounced hysteresis [4,5]. Capacitive pressure sensors usually struggle to achieve both high sensitivity and a wide linear detection range [6,7]. Piezoelectric pressure sensors are mainly sensitive to dynamic loads. Therefore, they are generally unsuitable for continuous monitoring of static pressure [8]. Triboelectric pressure sensors have excellent self-powered capabilities. However, their output stability can be affected by changes in ambient temperature and humidity [9].

To address these limitations, researchers have carried out extensive studies on the optimization of sensing mechanisms, high-performance functional materials, and multi- scale structural engineering. In particular, new flexible sensing materials have attracted considerable attention. These materials include conductive hydrogels, MXene-based composites, and carbon-based nanomaterials. Structural strategies, such as serpentine, three-dimensional porous, crack, wrinkle, and Kirigami designs, have also been widely introduced. These approaches can reduce local stress concentration during large deformation. They can also improve the sensitivity, linear response range, and environmental stability of flexible pressure sensors [10,11,12,13,14,15,16]. Meanwhile, the synergistic optimization of material systems and structural designs has further improved dynamic response, repeatability, and cycling stability [17,18]. This strategy provides new opportunities for the development of high-performance flexible pressure sensors. With the rapid progress of artificial intelligence and machine learning, flexible sensors are gradually evolving beyond conventional single-signal acquisition devices. They are becoming intelligent integrated systems that combine precise sensing, intelligent recognition, data communication, diagnosis, and treatment [19,20,21].

In recent years, researchers have systematically reviewed flexible pressure sensors from the perspectives of sensing mechanisms, functional materials, structural designs, fabrication methods, and applications. Xu et al. summarized recent progress in material design, device structures, fabrication technologies, and practical applications of high-performance flexible pressure sensors [2]. Zheng et al. further reviewed their performance evaluation metrics, sensing mechanisms, emerging materials, printing technologies, and application scenarios. They also discussed future development trends and challenges [22]. For specific sensing mechanisms, Mathew et al. focused on flexible capacitive pressure sensors and systematically summarized dielectric materials, structural designs, printing methods, and applications [23]. In terms of materials, Mohamadzade et al. reviewed the applications of carbon nanomaterials, metal nanomaterials, MXenes, and other two-dimensional nanomaterials in flexible pressure sensors [24]. Furthermore, Jiang et al. focused on ionogels as a specific material system and summarized their classification, fabrication methods, functional properties, and applications in flexible pressure sensors [25].

This review systematically summarizes recent progress in flexible pressure sensors and their intelligent applications. First, the working principles, performance characteristics, and suitable applications of typical sensing mechanisms are introduced and compared. These mechanisms include piezoresistive, capacitive, piezoelectric, triboelectric, iontronic, self-powered, and electrochemical sensing. Next, the development of key functional materials is summarized, including flexible substrates, carbon-based nanomaterials, metal nanostructures, conductive hydrogels, and MXenes. Particular attention is given to the effects of serpentine, porous, crack, wrinkle, and Kirigami structures on device flexibility, stretchability, sensitivity, and detection range. On this basis, representative applications of flexible pressure sensors are reviewed. These applications include physiological signal and human motion monitoring, disease diagnosis, machine-learning-assisted gesture recognition, human–machine interaction, and electronic skin. Finally, the remaining challenges and future research directions of flexible pressure sensors are discussed.

## 2. Literature Search Strategy

To provide a relatively comprehensive overview of recent advances in flexible pressure sensors, this review mainly focuses on studies published between 2020 and 2026. Some earlier studies with fundamental importance were also included when necessary. These studies provide background information on sensing mechanisms, functional materials, and structural designs. The literature search was mainly conducted using the Web of Science Core Collection database. “Flexible Pressure Sensor” was used as the Topic search term. The publication period was limited to 2020–2026. A total of 8316 records were obtained from the initial search. Based on the scope of this review, additional targeted searches were then conducted. These searches focused on specific sensing mechanisms, functional materials, structural designs, and application areas. Relevant references cited in representative research articles and recent review papers were also traced. This step was used to supplement important studies that were not covered by the initial search.

The literature was mainly screened according to its relevance to flexible pressure sensors and its contribution to the topics discussed in this review. Titles and abstracts were first examined to identify potentially relevant studies. Full texts were further examined when necessary. Priority was given to studies that directly investigated flexible pressure sensors. Studies that provided important theoretical or experimental evidence on sensing mechanisms, functional materials, structural designs, device performance, and typical applications were also included. Studies with limited relevance to flexible pressure sensing were not included in the main analysis. Highly repetitive studies and papers that did not provide substantial support for the topics discussed in this review were also excluded. In addition, some earlier studies with fundamental importance in material properties, structural design strategies, and sensing mechanisms were included through reference tracing.

After the initial database search, relevance-based screening, targeted supplementary searches, and reference tracing, a total of 143 references were selected for the analysis and discussion in this review. Recent review articles on flexible pressure sensors were also examined during the literature search. This helped us understand the scope and main focus of previous reviews and clarify the positioning of the present review. Representative recent reviews and their main research focuses have been discussed in the Introduction [2,22,23,24,25].

## 3. Sensing Mechanisms

Flexible pressure sensors are generally composed of flexible substrates and functional sensing materials. Their sensing mechanisms mainly include piezoresistive, capacitive, piezoelectric, and triboelectric sensing. In recent years, iontronic and electrochemical self-powered pressure sensing mechanisms have also attracted increasing attention. These mechanisms provide new approaches for detecting both static and dynamic pressures and achieving multifunctional integration. Table 1 summarizes flexible pressure sensors based on different sensing mechanisms and compares their performance reported in recent studies.

### 3.1. Piezoresistive Pressure Sensors

**Figure 1:**
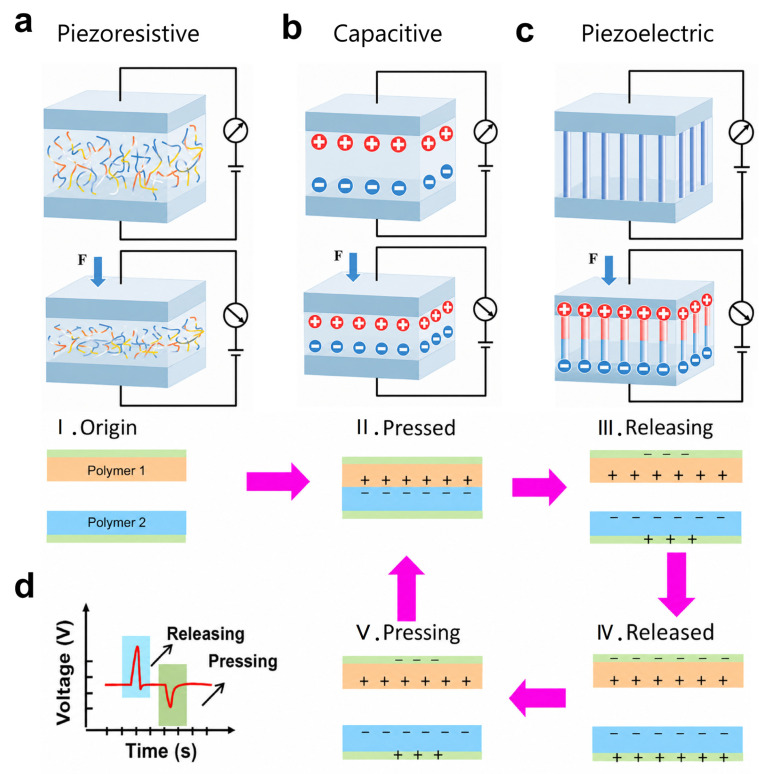
Schematic illustrations of the transduction mechanisms of (**a**) piezoresistive, (**b**) capacitive, and (**c**) piezoelectric flexible pressure sensors (reproduced with permission from [4]). (**d**) Working principle of a triboelectric nanogenerator (TENG) operating in the contact–separation mode (reproduced with permission from [32]).

Piezoresistive pressure sensors directly convert mechanical stimuli, such as strain or pressure, into changes in electrical resistance [4]. When an external mechanical force is applied to the sensor, the microstructure of the sensing material deforms. This deformation alters the conductive pathways, resulting in a change in the electrical resistance of the device [31]. By monitoring changes in the resistance signal, external pressure or deformation can be detected in real time, as shown in Figure 1a [4]. The resistance (R) of a conductor can be expressed as:

(1) $$R\;=\;\rho \frac{L}{A}$$

where ρ, L, and A represent the resistivity, length, and cross-sectional area of the conductor, respectively. When strain is applied, the change in resistance results from variations in both the geometric dimensions and the intrinsic resistivity of the material. The relative resistance change can be expressed as:

(2) $$\frac{\Delta R}{R}\;=\;\left(1\;+\;2v\right)\varepsilon \;+\;\frac{\Delta \rho }{\rho }$$

where v is the Poisson’s ratio, ε is the applied strain, and Δρ/ρ represents the relative change in resistivity associated with the piezoresistive effect. For conventional metallic piezoresistive materials, such as metal alloys, the resistance variation is primarily caused by geometric deformation, while the contribution from resistivity changes is relatively small. In contrast, semiconductor materials such as doped silicon exhibit a much stronger piezoresistive effect due to strain-induced changes in resistivity. Although conventional rigid piezoresistive materials exhibit stable electrical properties, their limited stretchability and nonlinear response under large deformation restrict their application in flexible sensing systems [5].

**Figure 2:**
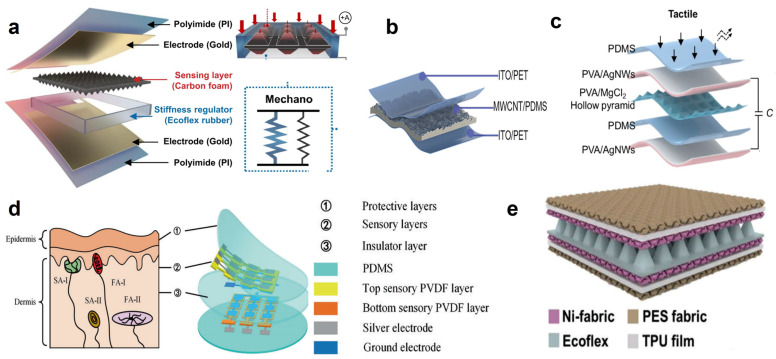
(**a**) Exploded view illustrating the design layout of the DPyCF@SR sensor (reproduced with permission from [35]). (**b**) Schematic of a spontaneously wrinkled capacitive sensor (reproduced with permission from [7]). (**c**) Schematic illustration of a flexible capacitive pressure sensor based on hollow micro-pyramid dielectric structures (reproduced with permission from [36]). (**d**) Schematic of a skin-inspired piezoelectric tactile sensor array (reproduced with permission from [26]). (**e**) Schematic of a TENG sensor with a pyramidal microstructure (reproduced with permission from [9]).

Piezoresistive sensors have been widely used in health monitoring, human motion detection, personal healthcare, human–machine interfaces, and electronic skin because of their simple and reliable operating principles, mature fabrication processes, simple readout mechanisms, high linearity, low power consumption, and potential for high pixel density. Compared with conventional rigid devices, the performance of flexible piezoresistive sensors is closely related to the conductive networks formed by the sensing materials and their deformation under pressure. Therefore, material selection and microstructural design are key strategies for improving sensitivity, sensing range, and mechanical stability [4]. Carbon-based materials such as carbon nanotubes and graphene exhibit good electrical conductivity and mechanical flexibility, enabling the formation of conductive networks on flexible substrates and resistance changes through pressure-induced variations in contact resistance and tunneling resistance [33,34]. In addition to material selection, microstructural design can further regulate the deformation and conductive pathways of the sensing layer, thereby amplifying pressure-induced resistance changes and balancing sensitivity and sensing range. As shown in Figure 2a, Chen et al. developed a flexible piezoresistive pressure sensor based on a double-sided pyramidal carbon foam (DPyCF) array and an elastic stiffness-regulating structure. The double-sided pyramidal and porous structures enhanced pressure-induced deformation and conductive pathway changes, while the stiffness-regulating structure improved load distribution under high pressures, enabling both high sensitivity and a wide linear sensing range [35].

However, piezoresistive sensors still suffer from hysteresis, temperature drift, and insufficient long-term stability. These limitations mainly arise from the viscoelastic behavior of flexible polymer substrates, irreversible changes in conductive networks during repeated loading and unloading, and temperature-induced variations in electrical conductivity and charge-carrier transport. The reliability and practical applicability of these devices can be further improved by constructing stable conductive networks, optimizing interfacial bonding between materials, and introducing temperature compensation and signal calibration strategies.

### 3.2. Capacitive Pressure Sensors

A capacitive pressure sensor consists of two parallel metal electrodes and a dielectric layer placed between them, as shown in Figure 1b [4]. Its sensing mechanism is based on the conversion of applied pressure into a change in capacitance. According to the ideal parallel-plate capacitor model, the capacitance (C) can be calculated as follows:

(3) $$C\;=\;\frac{\varepsilon S}{4\pi \mathrm{kd}}$$

where ε is the relative permittivity, k is the Coulomb constant, S is the electrode overlap area, and d is the separation distance between the two electrodes.

Under applied pressure, the dielectric layer is compressed or deformed, which changes the distance between the electrodes, the effective contact area, and the dielectric properties, thereby altering the capacitance. Capacitive pressure sensors have a simple structure, low power consumption, fast response, and good temperature stability. Therefore, they are widely used in flexible tactile sensing [37]. However, capacitive pressure sensors still suffer from small capacitance changes and are susceptible to parasitic capacitance and electromagnetic interference. This is mainly because the pressure-induced capacitance change in flexible devices is usually small. Meanwhile, interconnects, electrode edge effects, and packaging structures introduce additional parasitic capacitance, which reduces detection accuracy. Therefore, the interference immunity and detection performance of these devices can be improved by optimizing the electrode structure, using differential measurement methods, designing shielding structures, and constructing sensing layers with a high dielectric response [38].

To further improve the sensitivity and detection range of flexible capacitive pressure sensors, researchers usually optimize the dielectric layer and introduce special mechanical structures to enhance pressure-induced capacitance changes. The capacitance response mainly arises from changes in electrode spacing, effective area, and dielectric properties. Therefore, improving the deformability of the sensing layer can effectively amplify the pressure-induced capacitance response. As shown in Figure 2b, Lv et al. developed a flexible capacitive pressure sensor using a spontaneously wrinkled MWCNT/PDMS composite dielectric layer. The wrinkled structure provides more compressible space within the dielectric layer. This produces a more pronounced thickness change under pressure and thus enhances the capacitance response. Meanwhile, the addition of MWCNTs improves the dielectric properties of the composite, giving the device high sensitivity and good linearity in tactile sensing [7]. In addition, Xiong et al. developed a flexible capacitive pressure sensor with hollow micro-pyramid structures, as shown in Figure 2c. By introducing highly porous cavities inside the micro-pyramids, the dielectric layer exhibited greater compressive deformation under applied pressure while effectively delaying structural stiffening, thereby maintaining high sensitivity over a relatively wide pressure range. The sensor achieved a sensitivity of 3.45 kPa^−1^ in the range of 0–0.6 kPa and a detection limit of 0.21 Pa, while also exhibiting fast response and good cycling stability [36].

### 3.3. Piezoelectric Pressure Sensors

Piezoelectric pressure sensors convert mechanical stimuli into electrical signals by exploiting the ability of piezoelectric materials to generate polarization charges under mechanical stress. When external pressure is applied to a piezoelectric material, its internal crystal lattice deforms. This causes a displacement between the centers of positive and negative charges, generating a measurable potential difference across the material, as shown in Figure 1c [4]. The output signal of a piezoelectric sensor mainly depends on the polarization state of the material, variations in mechanical stress, and the processes of charge generation and transport. Commonly used piezoelectric materials include inorganic piezoelectric ceramics, such as PZT, and organic piezoelectric polymers, such as PVDF. PZT exhibits a relatively high piezoelectric response, whereas PVDF is more suitable for wearable sensing applications because of its flexibility and good mechanical properties [39].

Owing to their self-powered capability, high sensitivity, and fast response, piezoelectric sensors have broad application potential in dynamic pressure detection, vibration monitoring, and wearable electronic devices [40]. However, the output of piezoelectric sensors depends on variations in mechanical stress. The internally generated charges are also subject to charge leakage and dipole relaxation. Therefore, conventional piezoelectric sensors are generally unable to detect static pressure over long periods. To address this limitation, Chen et al. used a nanowire/graphene heterostructure to enhance interfacial charge retention. This design enabled the piezoelectric sensor to respond effectively to static pressure, thereby overcoming the limitation of conventional piezoelectric devices that are mainly suitable for dynamic signal detection [8]. In tactile sensing applications such as electronic skin, it is also important to distinguish different types of external stimuli in real time. Inspired by human skin, Lin et al. developed a piezoelectric tactile sensor array composed of a multilayer structure and a row–column electrode configuration. As shown in Figure 2d, the sensor array was integrated with a signal processor and logic algorithms. This system could detect and distinguish various external stimuli in real time [26].

### 3.4. Triboelectric Pressure Sensors

Triboelectric pressure sensors are self-powered sensors based on the coupling of contact electrification and electrostatic induction. When two materials with different electron affinities come into contact, electrons are transferred across the interface, generating triboelectric charges. External pressure then changes the contact area, separation distance, or relative position of the two triboelectric layers. These changes alter the internal electric field distribution and produce measurable voltage or current signals in the external circuit [41]. A typical triboelectric nanogenerator (TENG) consists of triboelectric materials, electrodes, and supporting substrates. According to their structural configurations, TENGs can be divided into vertical contact–separation mode, lateral sliding mode, single-electrode mode, and freestanding triboelectric-layer mode [42]. Because these modes share similar operating principles, the contact–separation mode is used here as an example to explain the working mechanism of a TENG. In the initial state, no induced charges are present on the electrodes, as shown in Figure 1(dI). When the surfaces of two different materials come into physical contact, triboelectric charges are generated on the contacting surfaces, as shown in Figure 1(dII). Once the two surfaces begin to separate, a potential difference is established, causing electrons to flow instantaneously from the bottom electrode to the top electrode, as shown in Figure 1(dIII). Electrostatic equilibrium is eventually reached when the two surfaces are completely separated, as shown in Figure 1(dIV). When the two surfaces are pressed together again, the electrostatically induced charges flow back through the external load to compensate for the potential difference, as shown in Figure 1(dV). The current signal generated throughout the entire process is shown in the lower-left corner of Figure 1d [32].

TENGs have several advantages, including environmental friendliness, low cost, structural diversity, light weight, and a wide range of material choices. However, their triboelectric output strongly depends on the surface charge state of the materials. Environmental humidity, temperature, and surface contamination can affect both charge generation and charge retention, resulting in reduced output stability. Therefore, improving the charge retention capability of triboelectric materials, optimizing encapsulation structures, and constructing stable surface microstructures are key strategies for enhancing the long-term reliability of these devices. As shown in Figure 2e, Zhang et al. developed a textile-based triboelectric sensing system. A narrow-gap encapsulation structure was constructed through a heat-sealing process, and a pyramidal array was introduced into the negative triboelectric layer. TPU-coated fabric was also used for encapsulation. This design improved the stability of the triboelectric output and enabled gait analysis and waist motion capture [9].

### 3.5. Iontronic Self-Powered Pressure Sensors

According to the ion transport process and electrical signal generation mechanism, iontronic self-powered pressure sensors can be classified into potentiometric, piezoionic, and ion-gradient types. All three types use ionic media to convert mechanical stimuli into electrical signals. However, their signal generation mechanisms are distinct.

Potentiometric sensors mainly detect pressure by using mechanical stimuli to modulate the equilibrium potential of an electrode–electrolyte system. These devices usually consist of ionic media and electrodes with different interfacial potential responses. When pressure is applied to the sensing layer, the state of the electrolyte, ion distribution, and electrode–electrolyte interface change. These changes alter the potential difference between the two electrodes. Wu et al. developed a potentiometric mechanoelectrical conversion device using a Prussian blue/carbon electrode and an Ag/AgCl electrode. Mechanical deformation regulated the electrode–electrolyte interface and converted mechanical stimuli into potential signals. This work provided a new sensing mechanism for iontronic electronic skin [27]. Subsequently, Lei et al. developed an ion-channel-induced self-powered flexible pressure sensor. Pressure changed the structure and transport state of the ion channels, thereby altering the output potential. The device had a wide pressure detection range from 0.7 Pa to 1300 kPa. It generated an open-circuit voltage of 0.58 V and a short-circuit current density of 3.2 μA cm^−2^. It also retained 95% of its performance after 5000 cycles and enabled both static and dynamic pressure detection [43].

Piezoionic sensors mainly generate electrical signals through mechanically induced nonequilibrium ion migration and space-charge separation. When a solvent-infused ionic gel, such as an ionic hydrogel or ionogel, is subjected to localized pressure or nonuniform deformation, a stress or hydrostatic pressure gradient can develop within the material. This gradient drives the migration of mobile ions. Different ions vary in size, mobility, and interactions with the polymer network. As a result, cations and anions migrate to different extents, creating a local charge imbalance and generating a current or potential output at the electrodes. Dobashi et al. demonstrated that hydrogels can generate ionic currents under mechanical pressure and developed a soft piezoionic mechanoreceptor. The piezoionic current lasted from milliseconds to hundreds of seconds. Its charge output was significantly higher than that of conventional triboelectric and piezoelectric devices, providing an important basis for piezoionic pressure sensing [44]. To address solvent evaporation and liquid leakage in conventional gel systems, Shevtsov et al. developed an all-solid-state self-powered ionic pressure sensor using a cross-linked poly (ionic liquid). The device detected pressure over a range of 0–80 kPa, generated an output voltage of approximately 190 mV, and exhibited response and recovery times of approximately 0.2 s. This design improved the long-term stability of the device [28].

Ion-gradient sensors use a pre-established humidity or ion-concentration gradient within the device to generate a driving force. In the absence of external pressure, ions migrate from regions of high chemical potential to regions of low chemical potential, generating an output signal. Under applied pressure, material deformation further changes the ion gradient, transport pathways, and internal resistance of the device, thereby modulating the output current or voltage. Huang et al. established an ion gradient using a high-humidity LiCl filter paper layer and a low-humidity carbon-nanotube filter paper layer. They combined this gradient with pressure-induced structural changes to regulate ion transport and achieve self-powered pressure detection. The device produced pressure-dependent current signals over a range of 0.1–100 kPa and was used for applications such as respiratory state recognition [45]. However, ionic sensors remain susceptible to variations in temperature and humidity, ion diffusion, and solvent instability. Therefore, developing stable ionic systems, solid-state electrolytes, and high-barrier encapsulation structures is essential for improving their long-term reliability.

### 3.6. Electrochemical Pressure Sensors

Electrochemical pressure sensors mainly include primary-battery-type and rechargeable-battery-type devices. Primary-battery-type devices generate electrical potential or current through spontaneous redox reactions in electrode–electrolyte systems. Mechanical pressure is converted into electrical signals by regulating the electrode–electrolyte contact state, ion transport, interfacial charge transfer, and internal resistance of the device. Rechargeable-battery-type devices generate output signals through reversible electrochemical reactions. The sensing signals are modulated by pressure-induced changes in the internal structure or interfacial impedance. Unlike piezoelectric sensors, which rely on crystal polarization, and triboelectric sensors, which depend on contact electrification, electrochemical pressure sensors generate potential or current outputs through electrochemical reactions. This mechanism can enable both static and dynamic pressure detection.

However, electrochemical pressure sensors still face challenges related to service life, flexibility, output power, and structural integration. To meet the requirements for long-term operation, Zhang et al. developed a rechargeable zinc-ion battery-type pressure sensor. An isolation layer was introduced into the solid-state zinc-ion battery structure, allowing pressure to regulate the output signal by changing the internal contact state and ion transport resistance. This device integrated pressure sensing with electrochemical energy storage. It achieved pressure detection over a range of 2 Pa–368 kPa and exhibited good charge–discharge performance and cycling stability [46]. It should be noted that this device requires precharging before operation. Therefore, it belongs to a battery-type pressure sensor powered by an internal energy storage unit.

To achieve flexible static and dynamic pressure detection, Kim et al. developed a flexible electrochemical pressure sensor composed of graphite/PDMS electrodes, an aluminum electrode, and a PDMS sponge coated with a hydrogel electrolyte containing water, glycerol, and NaCl. Under applied pressure, deformation of the sponge structure changes the electrode–electrolyte contact state, thereby regulating the electrochemical output. The device achieved a pressure detection range of up to 110 kPa and enabled self-powered safety alarms, glove-based control, and Morse code recognition without an external power supply [29]. To address the complex fabrication processes and high costs of conventional electrochemical pressure sensors, Huang et al. developed a paper-based electrochemical pressure sensor using Cu/Al electrodes, a LiCl electrolyte, and filter paper with a rough microstructure. Under pressure, the filter paper structure is compressed, enhancing contact between the electrodes and the electrolyte and thereby regulating the electrochemical reaction process. The device generated pressure-dependent current signals over a range of 1–30 kPa and achieved a maximum output current of 29.4 μA at 30 kPa. It was further applied to joint motion monitoring, respiration detection, and Morse code recognition [47]. Subsequently, Zhang et al. developed a high-power electrochemical pressure sensor using Mg/Cu electrodes and LiCl/PVA/CNT-modified filter paper. The device achieved pressure detection over a range of 0.6–100 kPa and generated an output current of 6 mA and a maximum power of 2.75 mW at 100 kPa. It could directly power devices such as LEDs and buzzers, while its output current could also be measured using an ammeter. The device was also used for respiration pattern recognition and infant sleep safety monitoring [30].

Regarding device structure optimization, Han et al. replaced the traditional sandwich structure with coplanar Cu/Zn electrodes and combined them with LiCl/PVA-modified filter paper to construct a flexible electrochemical pressure sensor. This design reduced the device thickness and improved its integration capability. The sensor achieved pressure detection over a range of 0.4–100 kPa while maintaining a broad response range and high sensitivity [48].

## 4. Materials for Flexible Sensors

The performance of flexible pressure sensors strongly depends on the selection of material systems. According to their functional roles, materials used to construct flexible pressure sensors mainly include flexible substrates, conductive nanomaterials, metallic materials, ionically conductive materials, and MXenes. These materials enable efficient pressure signal conversion by providing mechanical flexibility and regulating electronic transport, ion migration, and interfacial responses.

### 4.1. Flexible Substrates

Compared with traditional rigid materials such as metals, glass, and ceramics, flexible materials are more suitable for the fabrication of flexible electronic devices because of their excellent flexibility and deformability. Among various flexible substrates, polymers are widely used because of their low cost, light weight, good biocompatibility, and ease of integration with other functional materials. Common flexible substrates include polydimethylsiloxane (PDMS), polyimide (PI), Ecoflex silicone rubber, polyethylene terephthalate (PET), polyvinyl alcohol (PVA), polyacrylamide (PAAm), and polyethylene naphthalate (PEN).

Among these materials, PDMS is one of the most widely used elastomers because of its optical transparency and flexible siloxane-based polymer structure. It can be easily processed into micro- and nanostructures through techniques such as soft lithography and also exhibits good biocompatibility [49]. PDMS has an elastic modulus of approximately 0.4–3.5 MPa, indicating its relatively high deformability, and can withstand tensile strains of more than 40% [50]. PDMS, Ecoflex, and hydrogel substrates are suitable for skin attachment and physiological signal monitoring. TPU and textile substrates are more suitable for long-term wearable applications and motion monitoring. PI and PEN substrates are preferred for applications requiring high thermal or dimensional stability. The selection of substrates should also consider the deformation range, environmental conditions, and processing compatibility with subsequent sensing layers. Table 2 summarizes the fabrication methods, advantages, and major limitations of these typical substrate materials, providing guidance for the design and material selection of flexible sensors.

### 4.2. Carbon-Based Materials

Common carbon-based sensing materials used in flexible pressure sensors include carbon black (CB), carbon nanotubes (CNTs), graphene-based materials, including graphene, graphene oxide (GO), and reduced graphene oxide (rGO), and biomass-derived carbon materials. Carbon-based materials generally exhibit excellent electrical conductivity, mechanical properties, chemical stability, and compatibility with composite fabrication. They can achieve piezoresistive responses through changes in conductive pathways, interparticle spacing, and contact area under applied pressure.

Carbon black is an amorphous carbon material with a graphite-like microcrystalline structure. It has advantages including low cost, wide availability, simple processing, and good compatibility with polymer matrices [51]. When CB particles are dispersed in elastic substrates, they can form conductive networks near the percolation threshold. Under external pressure, the distance between particles decreases or new contact points are formed, resulting in changes in the electrical resistance of the composite. Compared with CNTs and graphene, CB has relatively lower electrical conductivity and a weaker ability to construct long-range conductive networks. However, it offers significant advantages in terms of cost and large-scale processing. Chen et al. prepared a conductive ink by solution blending SEBS, TPU, CB, and cellulose nanofibers and then coated it onto elastic fabrics. The resulting conductive coating exhibited good interfacial adhesion and mechanical stability [52]. CB can also be combined with CNTs, graphene, or MXene to construct multiscale conductive networks, thereby improving sensitivity, stability, and adhesion between the sensing layer and the substrate.

Carbon nanotubes (CNTs) are widely used one-dimensional carbon materials in flexible pressure sensors, mainly including multi-walled carbon nanotubes (MWCNTs) and single-walled carbon nanotubes (SWCNTs) [53,54]. Owing to their high aspect ratio, excellent electrical conductivity, and mechanical flexibility, CNTs can form continuous conductive networks at low loading levels. Under pressure, the distance between CNTs decreases, and the number of contact points increases, reducing contact resistance and tunneling resistance and generating a clear resistance response [55]. However, strong van der Waals interactions between CNTs can cause aggregation, which limits their uniform dispersion in polymer matrices. Common solutions include surface functionalization, the addition of dispersants, and the combination of CNTs with graphene or cellulose [56].

**Figure 3:**
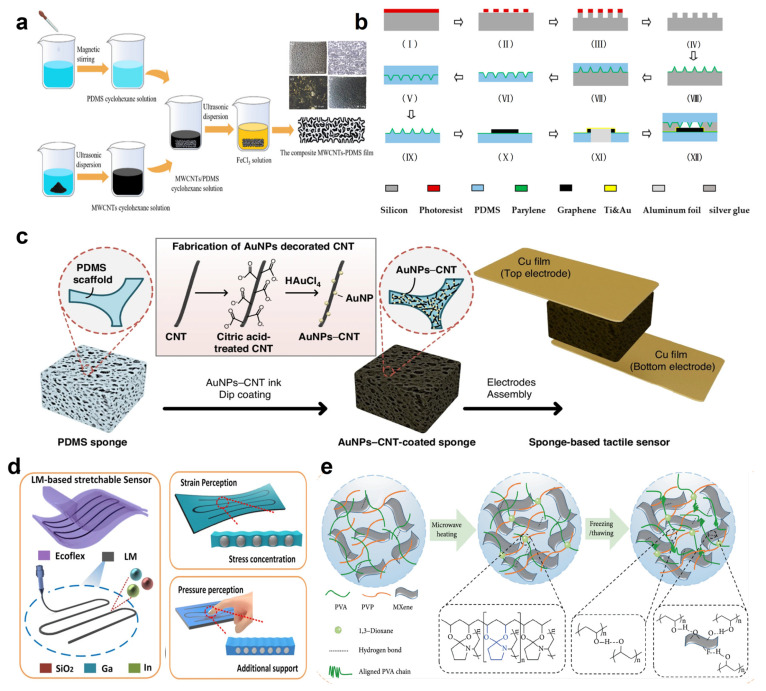
(**a**) Fabrication process of an MWCNT–PDMS film (reproduced with permission from [57]). (**b**) Fabrication process of a PDMS/bilayer graphene pressure sensor based on conical microstructures (reproduced with permission from [58]). (**c**) Schematic illustration of the fabrication process of an AuNPs–CNTs-coated sponge tactile sensor. The tactile sensor consists of an AuNPs–CNTs-coated sponge and copper electrodes (reproduced with permission from [59]). (**d**) Schematic illustration of a printable liquid-metal (LM) sensor integrated into a boxing glove (reproduced with permission from [60]). (**e**) Schematic illustration of the synthesis route of an MXene composite double-network hydrogel (reproduced with permission from [61]).

CNTs are often introduced into foams, sponges, and porous elastomers to increase compressible space and conductive contacts. CNT films can also be designed with micropores, microcracks, and wrinkles to enable dynamic reconstruction of conductive pathways. As shown in Figure 3a, Liao et al. fabricated a three-dimensional porous MWCNT/PDMS piezoresistive pressure sensor using porous nickel foam as a sacrificial template. The porous structure increased the compressible space and promoted the formation of conductive pathways during loading. The sensor achieved a pressure range of 0–50 kPa, a sensitivity of 0.487 kPa^−1^ at 20–35 kPa, and response and recovery times of 87 and 66 ms, respectively. It also successfully detected pulse signals before and after exercise [57].

Graphene is a two-dimensional material composed of sp^2^-hybridized carbon atoms. It exhibits a high specific surface area, high carrier mobility, excellent mechanical strength, and good chemical stability [62]. Under external pressure, changes in the contact area, interlayer spacing, and conductive pathways between graphene sheets generate resistance responses. However, graphene sheets are prone to restacking, and their intrinsic compressibility is limited. Therefore, porous structures, wrinkles, and microstructured networks are usually introduced to improve their deformation capability under pressure. Graphene is also commonly combined with PDMS, PU, PANI, PVA, and MXene to enhance conductive networks and interfacial stability [63].

Graphene oxide (GO) contains abundant oxygen-containing functional groups, providing good dispersibility and interfacial bonding capability. However, its electrical conductivity is relatively low. Reduced graphene oxide (rGO) removes some of these oxygen-containing groups and partially restores electrical conductivity, making it more suitable for constructing pressure-sensitive conductive networks. Cheng et al. designed a conical microstructured PDMS/bilayer graphene flexible pressure sensor, which achieved sensitivities of 0.122 ± 0.002 and 0.077 ± 0.002 kPa^−1^ in the ranges of 0–5 kPa and 5–20 kPa, respectively, as shown in Figure 3b [58]. The conical microstructures enhanced local deformation and changes in contact area under low pressure, thereby improving the pressure response of the sensor.

### 4.3. Metallic Materials

The selection of conductive materials and their interfacial bonding with flexible substrates are key factors determining the sensitivity, detection range, and cycling stability of flexible pressure sensors [64]. Metallic materials exhibit high electrical conductivity, ease of patterning, and diverse structural forms. They are commonly used as pressure-sensitive layers or flexible electrodes in forms such as metal nanowires, metal nanoparticles, metal films, and liquid metals [64,65].

Metal nanoparticles can improve the pressure response by increasing the number of conductive contact points and reducing interfacial contact resistance. Sim et al. deposited gold nanoparticles onto carbon nanotube surfaces and incorporated them into a three-dimensional porous PDMS substrate. As shown in Figure 3c, the porous structure was compressed under pressure, increasing the contact area and the number of conductive pathways between AuNPs and CNTs. This enabled the sensor to achieve both high sensitivity and a wide pressure detection range, making it suitable for pulse and plantar pressure monitoring [59]. The three-dimensional porous PDMS sensor coated with AuNPs/CNTs achieved a sensitivity of 23.23 kPa^−1^ in the low-pressure region and a detection range of up to 1125 kPa.

Liquid metals have unique advantages in deformable flexible electronics because of their excellent fluidity and high electrical conductivity [66]. Among them, gallium (Ga)-based liquid metals have become promising flexible conductive materials because of their high electrical conductivity, low melting point, and good flowability [67]. Ahn et al. encapsulated eutectic gallium–indium (EGaIn) liquid metal in Ecoflex microchannels. The device achieved continuous pressure sensing by detecting pressure-induced changes in the microchannel cross-sectional area and the resistance of the liquid metal. It was further applied to plantar pressure and gait monitoring [68]. As shown in Figure 3d, Qiu et al. introduced SiO_2_ particles into liquid metal to regulate its rheological properties. They developed a directly printable liquid-metal composite pressure sensor and integrated it into a tactile glove for recognizing punching force and motion states [60]. However, liquid metals still face challenges such as high surface tension, limited patterning accuracy, and potential leakage from encapsulation. These issues require further improvement through particle incorporation, microchannel design, and reliable encapsulation strategies.

### 4.4. Hydrogels

Hydrogels are three-dimensional hydrophilic polymer networks constructed from natural or synthetic polymers. They have excellent water absorption capacity and can absorb and retain large amounts of water or biological fluids without dissolving [69]. Owing to their high water content, porous structure, and soft mechanical properties, hydrogels can closely mimic the physicochemical properties of natural tissues and exhibit greater similarity to biological tissues than many synthetic biomaterials [70]. Hydrogels can rapidly swell in water while maintaining their original three-dimensional structures and retaining large amounts of water.

In recent years, significant progress has been made in the development of high-strength and tough hydrogels, including double-network hydrogels [71,72], nanocomposite hydrogels [73,74], micelle-crosslinked hydrogels [75], and polyzwitterionic hydrogels [76]. Through structural design and control of synthesis conditions, the mechanical properties of hydrogels, such as toughness, stretchability, and flexibility, can be precisely tuned to meet the requirements of flexible wearable devices [77]. The mechanical properties of hydrogels mainly depend on the polymer network structure and crosslinking density [78]. Meanwhile, their applications are strongly influenced by their physical properties, such as swelling degree, viscosity, elasticity, stiffness, and mechanical strength, as well as their chemical properties and microstructures [69].

Inspired by conventional hydrogel structures and conductive filler composites, conductive hydrogels can be fabricated by combining polymer networks for structural support with functional fillers for electrical conductivity [79,80]. Compared with flexible sensors based on filler/elastomer composites, conductive hydrogels offer advantages in biomimetic structures, functional properties, and biocompatibility [81]. Therefore, researchers have developed various multifunctional conductive hydrogels by introducing nanofillers or functional additives into hydrogel networks, enabling their widespread application in wearable sensing [82]. Significant progress has been made in conductive hydrogel-based wearable pressure sensors. These sensors can be attached to the skin for real-time monitoring of human motions and subtle physiological signals and can also be applied in human–machine interaction and electronic skin [83,84,85].

Lu et al. developed an MXene-based double-network conductive hydrogel for flexible pressure sensing. As shown in Figure 3e, MXene nanosheets formed continuous conductive networks inside the hydrogel. Under pressure, the hydrogel was compressed, increasing the contact between MXene sheets and causing a significant change in resistance. The sensor achieved a sensitivity of 10.75 kPa^−1^ below 13.8 kPa and 0.59 kPa^−1^ in the range of 13.8–61.5 kPa. It was used for touch sensing and human pressure signal detection [61]. In addition, Xiao et al. fabricated a pressure sensor based on natural locust bean gum hydrogel with a three-dimensional macroporous structure using rosin particles as a sacrificial template. The porous structure underwent significant compression under pressure, promoting the formation of conductive pathways and changes in the contact area between the hydrogel and electrodes. The sensor maintained high pressure sensitivity over the range of 0.1–100 kPa and enabled human motion monitoring [86].

### 4.5. MXenes

MXenes are a class of two-dimensional transition metal carbides, nitrides, and carbonitrides first reported in 2011. They are usually prepared by selectively etching the A elements from MAX phases. In the term “MXene,” “MX” indicates its origin from MAX-phase ceramics, while “ene” reflects its graphene-like two-dimensional layered structure [87]. MXenes exhibit excellent electrical properties, good flexibility, and tunable surface chemistry. However, they are prone to oxidation in air, which affects their long-term stability.

Ma et al. fabricated a flexible biodegradable pressure sensor based on MXene nanosheets using an immersion-drying method. By controlling the degree of MXene oxidation, the sensitivity of the sensor was tuned. The sensor achieved a sensitivity of 28.43 kPa^−1^ and could detect pressures as low as 0.8 Pa [88]. Although nanoscale monolayer MXene exhibits excellent mechanical properties, macroscopic MXene films often suffer from restacking due to interlayer interactions, resulting in reduced mechanical performance [89,90,91]. To address this issue, Cheng et al. introduced one-dimensional aramid nanofibers (ANFs). Hydrogen bonding between ANFs and MXene layers was used to construct high-strength MXene films, significantly improving their mechanical properties [12].

In addition, the abundant functional groups and active sites on MXene surfaces facilitate their integration with other materials [92,93]. Yang et al. fabricated a flexible pressure sensor based on MXene/PU/interdigitated electrodes using a spray-coating method. The spiny PU microstructure served as a flexible substrate, enabling an ultrahigh sensitivity of 509.8 kPa^−1^. Meanwhile, the hydrogen-bonding network in PU endowed the device with self-healing capability [17]. Inspired by the asymmetric structure of human skin, Bai et al. developed a PU-TA@MXene biomimetic Janus multifunctional sensor. The device achieved a fracture strain of 2056.67% and a tensile strength of 50.78 MPa. It could also selectively distinguish compression, bending, and stretching stimuli [94].

## 5. Structural Design of Flexible Sensors

For intrinsically rigid conductive materials, structural design is an important strategy for achieving macroscopic stretchability in flexible pressure sensors [95]. By introducing porous, wrinkled, cracked, and serpentine structures, local stress concentrations generated during deformation can be alleviated. These structures can also regulate changes in contact area and conductive pathways under pressure, thereby improving sensitivity, detection range, and stability [96,97]. In recent years, various innovative structural designs have been developed. This section mainly reviews five representative structures: serpentine, three-dimensional porous, crack, wrinkle, and Kirigami structures. Table 3 summarizes the material compositions, sensitivities, detection ranges, cycling stability, advantages, and limitations of flexible pressure sensors based on these five typical structures.

**Figure 4:**
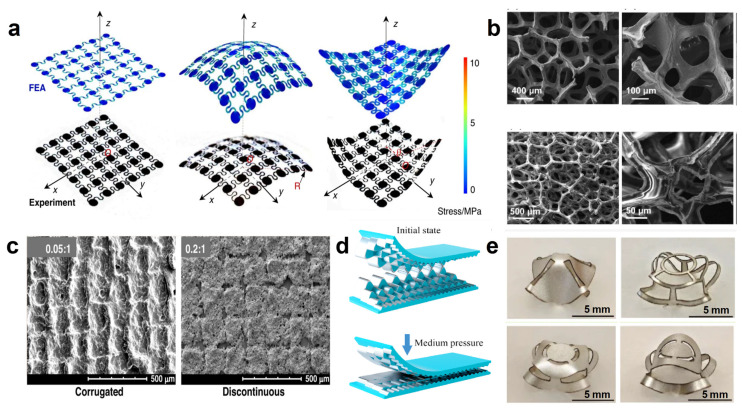
(**a**) Experimental results and three-dimensional finite element analysis of the stretchable ionic pressure sensor (SIPS) under three combined deformation modes (reproduced with permission from [18]). (**b**) SEM image of a three-dimensional interconnected graphene foam (reproduced with permission from [98]). (**c**) SEM images of NW-CNT/PDMS sensing films with different weight ratios (reproduced with permission from [14]). (**d**) Schematic diagrams of the sensor structure in the initial state and under medium pressure (reproduced with permission from [15]). (**e**) Four different three-dimensional Kirigami structures (scale bars: 5 mm) (reproduced with permission from [16]).

Serpentine structure: Serpentine structures consist of two semicircular segments connected by straight lines. They can rotate in plane or deform out of plane, thereby reducing local strain under tensile deformation [97]. Their stretchability originates from the extension of the wavy “two-dimensional spring” configuration [95]. In flexible pressure sensors, serpentine structures are commonly used to construct stretchable electrodes, interconnects, and sensor arrays. They allow sensing units to conform to human joints, skin, and other irregular surfaces while reducing the influence of substrate deformation on pressure signals. Xu et al. developed a stretchable ionic pressure sensor using serpentine PI/Au/carbon–graphite composite interconnect electrodes. As shown in Figure 4a, the device features serpentine electrodes integrated with an overall flexible structure. It tolerates a tensile strain of 20%; moreover, it achieves a sensitivity of 12.43 kPa^−1^ and linear detection range of up to 1 MPa, demonstrating that serpentine structures can improve stretchability and mechanical stability of pressure sensors [18].

3D porous structure: Three-dimensional porous structures are typically composed of interconnected pores in foams, sponges, or aerogels. They can reduce structural stiffness and enhance pressure responsiveness through pore-wall bending, pore collapse, and conductive pathway reconstruction [97]. As shown in Figure 4b, Li et al. developed a pressure sensor based on three-dimensional interconnected graphene foam. The sensor achieved a sensitivity of 0.42 kPa^−1^ within the range of 0–390 Pa, exhibited a wide detection range of up to 42 kPa, and showed a response time of 62 ms. It also maintained stable performance over 10,000 cycles [98]. Jia et al. fabricated a polyimide fiber/carbon nanotube aerogel pressure sensor with a gradient porous structure. The sensor achieved a wide detection range from 10 Pa to 1.58 MPa and a pressure resolution of 0.001% at 1 MPa [99]. These results demonstrate that regulating pore size and pore gradients can effectively balance the sensitivity, detection range, and stability of pressure sensors.

Crack structure: Crack structures are inspired by the slit sensilla of arthropods. For example, slit organs on the bodies of spiders can detect weak vibrations and changes in airflow [97]. Since Kang et al. introduced nanocracks into flexible sensors in 2014 [100], crack structures have been widely employed to amplify changes in conductive pathways induced by external forces. Their sensitivity is mainly determined by crack width, depth, and density [101]. As shown in Figure 4c, Wang et al. constructed periodic microcracks in an MWCNT/PDMS pressure-sensitive film using screen printing. Under compression, crack closure and increased contact between adjacent units enhanced the pressure response. The sensor achieved a sensitivity of 18.092 kPa^−1^ in the range of 0–10 kPa, a detection range of up to 400 kPa, response and recovery times of 41.8 and 15.4 ms, respectively, and stable performance over 50,000 cycles under a pressure of 150 kPa [14].

Wrinkle structure: Wrinkle structures are widely found in nature and are generally formed by bending deformation induced by nonuniform stresses, such as the wrinkles observed on human skin [97]. Wrinkle structures can release stress through unfolding and structural reconfiguration under external strain. This helps prevent the fracture or failure of functional materials and improves device stretchability and working range [96]. Wrinkled MXene films can be readily fabricated by depositing monolayer MXene sheets onto pre-stretched substrates. This strategy facilitates the large-scale fabrication of pure MXene films and enables pressure sensors to achieve excellent overall performance [102]. To further improve sensing performance, Liu et al. constructed gradient wrinkle structures on PDMS surfaces through ultraviolet ozone treatment and pre-stretching. AgNWs were subsequently sprayed onto the surface to form a conductive sensing layer, resulting in a flexible piezoresistive pressure sensor (Figure 4d). With increasing pressure, the gradient wrinkles progressively generated new contact points, thereby continuously increasing the effective contact area of the conductive layer. This structure enabled both high sensitivity and a wide detection range. The sensor achieved a sensitivity of 0.947 kPa^−1^ in the low-pressure region, detected pressures as low as 10 Pa, exhibited a detection range of 10–50 kPa, and was successfully applied to touch sensing, swallowing monitoring, and joint motion detection [15].

Kirigami structure: Kirigami structures originate from the traditional art of paper cutting. By introducing specific cutting patterns into two-dimensional films, planar structures can be transformed into complex three-dimensional configurations. This design enables materials to undergo large deformations in both linear and nonlinear directions. Meanwhile, the free rotation and buckling of individual cut units help prevent structural damage, providing an effective strategy for improving the stretchability of flexible sensors [97]. In practical applications, Kirigami structures are usually fabricated by introducing microscale cutting patterns into conductive films. Their mechanical properties can be precisely tuned by adjusting the geometric parameters of the cutting patterns [95]. As shown in Figure 4e, Zhang et al. fabricated Kirigami patterns on silver-coated PVDF films using femtosecond laser processing. Through compressive buckling induced by the release of a pre-stretched substrate, the two-dimensional precursor was transformed into a three-dimensional piezoelectric sensing structure. By adjusting the cutting pattern and the elastic modulus of the encapsulation material, the sensitivity of the sensor could be tuned from 0.025 to 0.056 V·N^−1^. The maximum sensitivity was approximately four times that of a planar PVDF sensor. In addition, the sensor maintained stable output over 6000 loading cycles under a pressure of 1356 kPa [16].

Overall, the five structural designs exhibit distinct performance trade-offs. Serpentine and Kirigami structures mainly improve mechanical deformability and conformability, whereas porous, crack, and wrinkle structures primarily enhance pressure-sensing performance by regulating structural compression, conductive pathways, or contact-area variation. Therefore, the selection of a structural design should be guided by the target application, with particular consideration of the required sensitivity, detection range, stretchability, and mechanical stability.

## 6. Applications of Flexible Pressure Sensors

Flexible pressure sensors have a wider range of applications than conventional pressure sensors because of their excellent flexibility, stretchability, and ability to conform to the human body and irregular surfaces. They have been widely applied in areas such as motion monitoring, healthcare, human–machine interaction, and smart wearable devices [103]. Different applications require different sensor performance characteristics, including sensitivity, detection range, response speed, and stability. This section focuses on the typical applications of flexible pressure sensors in motion monitoring, healthcare, and human–machine interaction.

### 6.1. Motion Monitoring

Flexible pressure sensors have been widely used for human motion monitoring. They can detect both subtle physiological signals, such as facial expressions, respiration, and pulse, and large body movements, such as finger bending and elbow motion [104]. By sensing changes in pressure or contact force caused by body movements, these sensors convert mechanical deformation into electrical signals for motion recognition and analysis. Developing sensors with high flexibility, sensitivity, and a wide detection range is essential for improving monitoring accuracy.

Liu et al. developed an Al–Al_2_O_3_–CB capacitive pressure sensor with a sensitivity of 24.62 kPa^−1^ and a response time of 33 ms for physiological signal monitoring and motion detection [105]. Xiong et al. proposed a highly sensitive capacitive pressure sensor with a sensitivity of 30.2 kPa^−1^ under pressures below 130 Pa [106]. Chen et al. developed a skin-inspired piezoresistive pressure sensor capable of detecting various human motions, including finger pressing, facial movements, walking, and running. It could also recognize handwritten letters based on differences in writing force [107].

Innovations in materials and fabrication methods have further improved sensor performance. Chen et al. fabricated a TPU/PDA/MXene conductive composite foam using directional freezing and dip-coating methods. The resulting pressure sensor exhibited high sensitivity and could monitor facial, hand, and foot movements, with potential applications in smart shoes [108]. In addition, the integration of flexible sensors with smart textiles provides new opportunities for wearable sports monitoring. Tang et al. developed a smart sports bandage that could detect movements of multiple body parts and assist in sports training [109]. Khandakar et al. developed a wearable smart insole system for real-time plantar pressure monitoring, gait analysis, and foot health assessment, providing a portable solution for home rehabilitation [110]. Plantar pressure analysis has also been applied to gait and rehabilitation assessment in patients with stroke, Parkinson’s disease, and sports injuries by analyzing pressure distribution, support time, and center-of-pressure trajectories [111].

Flexible sensors for human motion monitoring still face challenges in sports training applications. For example, sensors used in underwater or extreme environments require excellent waterproof and antifreezing properties. Sun et al. developed a superhydrophobic Hf–SiO_2_ functional coating that provided self-cleaning capability, corrosion resistance, and environmental stability. The resulting waterproof wearable sensor enabled full-range monitoring of human motion underwater [112]. For low-temperature environments, hydrogels have become promising sensing materials because of their unique properties. Nie et al. developed a hydrogel-based sensor capable of monitoring human motion at −30 °C [113]. Yu et al. reported an XG-Fe^3+^/PAAm-Gl organohydrogel sensor that maintained motion-monitoring performance at −40 °C [114]. Li et al. developed a hydrogel-based flexible sensor that could monitor human postures under extreme temperatures or in outdoor environments for up to 30 days. It could also detect acoustic signals [115].

### 6.2. Medical and Healthcare Systems

Cardiovascular disease (CVD) refers to a group of disorders affecting the heart and vascular system. Continuous physiological monitoring using wearable sensors provides new approaches for the early screening, assisted diagnosis, and health management of CVD [116]. Blood pressure (BP) and heart rate are important indicators of cardiovascular function [117]. Clinically, cuff-based sphygmomanometers are commonly used to measure systolic blood pressure (SBP), diastolic blood pressure (DBP), and mean arterial pressure (MAP) [118]. However, repeated inflation and deflation of the cuff can cause discomfort, limiting its use for long-term continuous monitoring.

**Figure 5:**
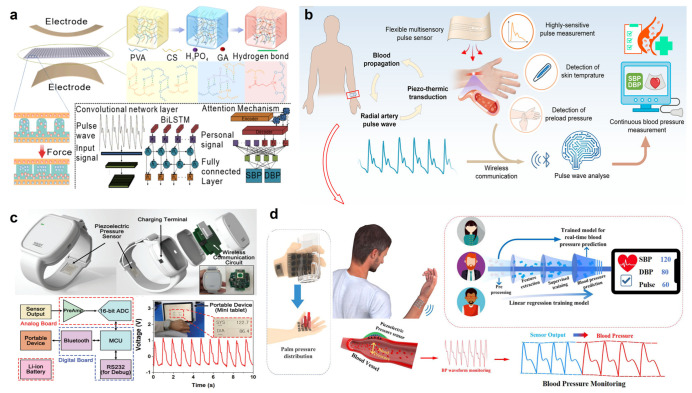
(**a**) Design process, sensing mechanism, and CNN-BiLSTM-Attention-BP model of a flexible capacitive pressure sensor based on ionic hydrogels (IHs) (reproduced with permission from [120]). (**b**) Working principle and blood pressure measurement scheme of a multimodal pulse sensor integrating pulse wave, skin temperature, and preload pressure sensing with machine-learning-based blood pressure estimation (reproduced with permission from [122]). (**c**) Schematic design of a WPBPS-embedded smartwatch, wireless communication circuit, and pulse waveforms transmitted from the smartwatch to a portable device via wireless communication (reproduced with permission from [123]). (**d**) A 4 × 4 sensor array for palm pressure distribution monitoring and wrist pulse wave acquisition, together with blood pressure prediction based on feature extraction and calibration using a mercury sphygmomanometer (reproduced with permission from [124]).

To address this issue, flexible pressure sensors have been developed to detect weak pressure changes caused by vascular pulsation. Combined with signal-processing methods and data-driven models, these sensors enable BP estimation. Bijender et al. developed a flexible capacitive pressure sensor based on polydimethylsiloxane (PDMS) and deionized water (DIW). A porous structure was introduced using baking powder to improve the sensing performance. The sensor achieved a sensitivity of 0.021 Pa^−1^, a detection limit of 1 Pa, and a response time of 100 ms. Tests on 160 subjects showed clear pulse wave signals, and the measured BP and pulse rate showed good agreement with those obtained using an OMRON electronic sphygmomanometer and a mercury sphygmomanometer, meeting relevant international standards [119]. Liu et al. further developed a flexible capacitive pressure sensor based on a multi-crosslinked dual-network ionic hydrogel. As shown in Figure 5a, the hydrogel was formed through physical and chemical crosslinking of polyvinyl alcohol, chitosan, phosphoric acid, and gum arabic. A uniform truncated-cubic microstructure was fabricated on the surface using a grid template. The sensor achieved a wide detection range of 0–290 kPa and a pressure resolution of approximately 1.3 Pa. It could detect weak wrist pulse signals and estimate BP by combining pulse wave features with a neural network model, demonstrating the potential of ionic hydrogel pressure sensors for intelligent BP monitoring [120]. Sun et al. proposed a BP monitoring system based on an improved arterial tonometry method. A parylene/PDMS flexible sensor was used to collect wrist pulse pressure signals, and a random forest model was applied to establish the relationship between pulse wave features and BP. Compared with traditional arterial tonometry, the system improved the measurement accuracy of SBP and DBP by 31.4% and 21%, respectively [121].

To reduce the effects of wearing conditions and individual differences on measurement accuracy, Tian et al. proposed a flexible multimodal pulse sensor, as shown in Figure 5b. The sensor can simultaneously measure radial artery pulse, skin temperature, and applied pressure. It achieved a pressure detection range of up to 228.2 kPa, a detection limit of 4 Pa, and a response time of 88 ms. Pressure and temperature signals were collected at 125 Hz and processed using a 30 s sliding window with a 5 s step size. After feature extraction in the amplitude, time, and frequency domains, 28 sensing features were combined with heart rate, age, height, weight, body mass index, and gender to form a 33-dimensional input vector. A multilayer perceptron with two hidden layers was then used to estimate SBP and DBP. Based on 260 datasets from 18 subjects, the errors in SBP and DBP were −0.03 ± 5.56 mmHg and 0.12 ± 3.91 mmHg, respectively, meeting the AAMI standard [122]. Min et al. integrated a wearable piezoelectric blood pressure sensor into a smartwatch. A PDMS passivation layer and a medical-grade adhesive layer were introduced to improve sensitivity and wearing stability. After filtering, amplification, and wireless transmission, the pulse signals were processed using a linear regression model for BP estimation. Compared with a commercial sphygmomanometer, the average differences in SBP and DBP were −0.89 ± 6.19 mmHg and −0.32 ± 5.28 mmHg, respectively, enabling portable continuous monitoring (Figure 5c) [123].

In addition to BP, heart rate and pulse waveforms provide important information about cardiac rhythm and peripheral vascular conditions. Chen et al. developed a breathable piezoresistive pressure sensor composed of MXene-modified polyester fabric and electrospun TPU nanofiber membranes, as shown in Figure 5d. The sensor achieved a sensitivity of 652.1 kPa^−1^, a detection range of 0–60 kPa, and response and recovery times of 36 and 20 ms, respectively. A 4 × 4 sensor array was further constructed to locate the region with the strongest pulse signal on the wrist. Combined with a flexible acquisition circuit, the system enabled signal processing, wireless transmission, and mobile display. It obtained stable radial artery pulse waves and measured a pulse rate of 90 beats/min, demonstrating its potential for wearable heart rate and pulse monitoring [124]. Flexible pressure sensors have evolved from simple pulse signal acquisition devices into integrated monitoring platforms combining signal processing, machine learning, and wireless communication. However, current systems still rely on external devices, and further improvements are needed in power consumption, latency, and long-term stability. Future research should focus on integrating sensors, front-end circuits, lightweight algorithms, and low-power processing units to achieve long-term continuous cardiovascular monitoring.

### 6.3. Human–Machine Interaction

Traditional devices used for human–machine interaction cannot fully satisfy users’ requirements for comfort and adaptability. Their rigid structures limit conformal contact with the human body and the development of more flexible and natural interaction technologies. Therefore, flexible sensors with excellent mechanical flexibility and stretchability provide a promising solution to these limitations [125]. Wearable sensor-based intelligent human–machine interaction has emerged as an important research trend. In this field, flexible pressure sensors are mainly applied in gesture recognition, interactive interfaces, and electronic skin systems [126].

#### 6.3.1. Gesture Recognition

Gesture recognition is a key technology in human–machine interaction. It enables more natural and intuitive communication between humans and computers by capturing, analyzing, and interpreting human gestures [127]. Traditional rigid sensors exhibit poor conformability to human skin, which can affect signal stability and accuracy. In contrast, flexible pressure sensors provide excellent conformal contact, offering new opportunities for comfortable and reliable wearable gesture recognition systems.

Wang et al. developed a flexible wearable gesture recognition system based on an iontronic capacitive pressure sensor array and a deep convolutional neural network. Through multichannel sensing, the high noise robustness of iontronic capacitive sensors, and feature extraction using the deep convolutional neural network, the system achieved recognition accuracies of 99.9%, 93.2%, and 93.1% under cross-trial testing, intra-session re-wearing, and electrode displacement conditions, respectively [21]. Hou et al. proposed a flexible porous phenyl silicone/functionalized carbon nanotube (PS/FCNT) pressure sensor with a cross-pore crack structure. Combined with a Transformer-based action recognition model, the system recognized six human actions with an overall accuracy of 94.96% [128].

**Figure 6:**
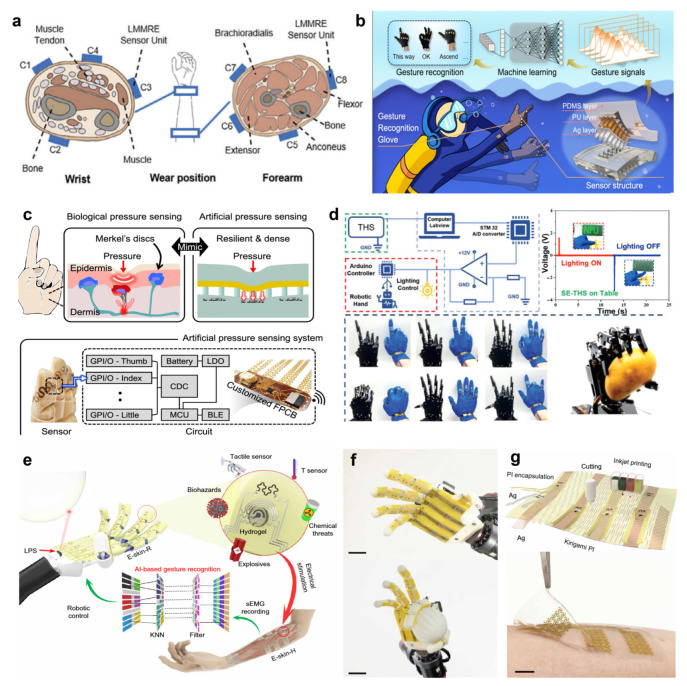
(**a**) Schematic diagram of sensor placement (end and middle of forearm) (reproduced with permission from [129]). (**b**) Schematic illustration of the biomimetic micropillar tactile sensor (MPTS)-based gesture recognition glove. The micropillar design is inspired by the tube feet of starfish, and the machine-learning-assisted gesture recognition process is illustrated (reproduced with permission from [130]). (**c**) Schematic illustrations of biological and artificial pressure sensing mechanisms and the corresponding artificial pressure sensing system (reproduced with permission from [131]). (**d**) Applications of the SE-THS sensor in human–machine interaction, including light control, human gesture recognition, and mango grasping (reproduced with permission from [132]). (**e**) Schematic diagram of M-Bot, consisting of two fully printed soft electronic skins: a human-skin interface and a robotic-skin interface for AI-based robot control and user interaction feedback based on multimodal physicochemical sensing (reproduced with permission from [133]). (**f**) Example of the robotic-skin interface (reproduced with permission from [133]). (**g**) Prototype schematic of the robotic-skin interface (reproduced with permission from [133]).

Chen et al. developed a flexible pressure-sensing bracelet based on a liquid metal-filled magnetorheological elastomer (LMMRE) and combined it with a support vector machine (SVM) for gesture recognition (Figure 6a). The system recognized seven gestures with offline and real-time accuracies of 98.2% and 92.04%, respectively, demonstrating its potential for real-time gesture recognition and prosthetic control [129]. Inspired by starfish tube feet, Liu et al. developed a waterproof gesture recognition glove based on flexible micropillar piezoresistive pressure sensors, as shown in Figure 6b. Ten sensing units were integrated at the finger joints to detect pressure changes during hand movements. Combined with a fully connected neural network, the system achieved an accuracy of 99.8% in recognizing 16 underwater gestures, expanding the applications of flexible pressure sensors in human–machine interaction under challenging environments [130].

#### 6.3.2. Interactive Interfaces

A human–machine interface (HMI) is an important electronic system that enables bidirectional information exchange between humans and machines. It converts human actions, touch inputs, and user intentions into machine-readable control signals and provides feedback from devices to users [125]. Flexible pressure sensors possess excellent flexibility, stretchability, and conformability, allowing them to convert mechanical stimuli caused by touch, pressing, joint bending, and muscle movements into electrical signals. Therefore, they have become important sensing units in wearable HMI systems [134,135]. Compared with traditional rigid sensors, flexible pressure sensors are more suitable for attachment to the skin or curved surfaces, enabling continuous acquisition of human motion information while improving wearer comfort.

However, flexible capacitive pressure sensors are often affected by water droplets, nearby conductive objects, device bending, and array crosstalk, resulting in baseline drift and false triggering. To address these issues, Yang et al. developed a flexible capacitive pressure sensor array with an electrode spacing of only 900 nm. As shown in Figure 6c, the device achieved a detection limit of 20 Pa, a response time of less than 46.8 ms, and stable responses over a pressure range of 0–330 kPa. It also maintained stable performance after 100,000 loading cycles at 100 kPa. In addition, the sensor achieved a spatial resolution of 11.89 units cm^−2^ and a low crosstalk level of 0.3% between adjacent units, enabling applications in wireless pressure monitoring and force-sensitive touch panels [131].

Beyond externally powered pressure sensors, triboelectric sensors can directly convert mechanical motions into electrical signals, providing new approaches for low-power and self-powered HMI systems [136]. Tao et al. developed a tactile sensor based on a micro-pyramid-structured double-network ionic organohydrogel, as shown in Figure 6d. The sensor exhibited a transparency of approximately 85%, a sensitivity of 45.97 mV Pa^−1^, a detection limit of 50 Pa, and a response time of about 20 ms. It maintained stable performance after 36,000 cycles. With a peak power density of 20 μW cm^−2^, the device could serve as a self-powered touch switch to control electronic devices and robotic hands through finger movements [132]. Flexible pressure sensors are driving the development of HMI systems from simple touch input toward high-sensitivity sensing, robust detection under interference, and closed-loop feedback. Future research should further improve weak-pressure detection, environmental adaptability, and long-term stability [137]. The integration of wireless communication, self-powered sensing technologies, and intelligent algorithms will enable more accurate, low-power, and reliable wearable HMI systems.

#### 6.3.3. Electronic Skin

Touch perception is an essential function of human skin for sensing the external environment. Receptors in the skin respond to stimuli such as pressure, stretching, bending, vibration, and temperature, allowing humans to perceive the shape, texture, and mechanical properties of objects [138]. Electronic skin (e-skin) is a flexible electronic system designed to mimic the sensory functions of human skin. Flexible pressure sensors serve as key components by converting mechanical stimuli, including touch, pressure, and impact, into electrical signals. Owing to their flexibility, stretchability, and conformability to curved surfaces, e-skins can be attached to the human body, prostheses, and robotic surfaces, showing broad application potential in health monitoring, human–machine interaction, and robotic tactile perception.

Yu et al. developed an AI-enabled M-Bot system based on fully printed stretchable electronic skin, as shown in Figure 6e–g. The human-side e-skin collected electromyographic signals for motion recognition, while the robot-side e-skin integrated tactile and environmental sensing functions for remote control and interactive feedback [133]. This work demonstrated that flexible pressure sensors can be combined with electrophysiological sensing and machine learning to construct e-skin systems integrating perception, recognition, and feedback.

In e-skin applications, iontronic pressure sensors based on the electric double-layer (EDL) effect exhibit high interfacial capacitance and can achieve both static and dynamic tactile sensing through pressure-induced changes in contact area [139]. Yang et al. developed a gradient micropyramid iontronic pressure sensor with a sensitivity of 33.7 kPa^−1^ over a range of 0–1700 kPa, a detection limit of 0.36 Pa, and a response time of 6 ms. The sensor was applied to pulse monitoring, robotic hands, and pressure-sensitive seats [140]. Zhang et al. proposed a bonded microstructured piezocapacitive sensor with a response time of approximately 0.04 ms, a detection limit of 0.007 Pa, and the ability to detect high-frequency vibrations up to 12.5 kHz, enabling dynamic tactile sensing [141]. Huang et al. constructed a sensor with a gradient modulus and microstructured design, achieving high sensitivity for weak touch, plantar pressure, and impact detection. Combined with machine learning, the sensor enabled handwritten identity recognition [142].

Advances in polymers, ionic gels, and conductive nanomaterials have further improved the performance of e-skins [143]. However, practical applications still face challenges, including the trade-off between sensitivity and detection range, mechanical interference, complex array wiring, and insufficient stability during long-term wear. Future research should focus on multiscale structural design, multimodal signal decoupling, and machine-learning algorithms to improve the accurate recognition of pressure magnitude, location, and dynamic characteristics.

## 7. Conclusions

Flexible pressure sensors have attracted extensive attention in wearable electronics, smart healthcare, electronic skin, and human–machine interfaces because of their flexibility, stretchability, and excellent compatibility with biological interfaces. Recent advances in functional materials, micro/nanofabrication, and flexible electronics have improved their sensitivity, detection range, stability, and functional integration. Various sensing mechanisms, including piezoresistive, capacitive, piezoelectric, and triboelectric sensing, together with advanced materials such as carbon-based materials, metal nanostructures, conductive hydrogels, and MXenes, have contributed to enhanced sensor performance. Meanwhile, structural designs, including porous, crack, wrinkle, and Kirigami structures, have further improved sensitivity, mechanical adaptability, and reliability.

Through the integration of sensing mechanisms, materials, and structural designs, flexible pressure sensors have enabled continuous monitoring of physiological and mechanical signals and have been widely applied in health monitoring, gesture recognition, electronic skin, and intelligent human–machine interaction. Future research should focus on improving long-term stability, environmental adaptability, multimodal sensing capabilities, and scalable fabrication. Flexible pressure sensors are expected to evolve toward highly integrated, intelligent, and multifunctional systems for next-generation wearable electronics and smart healthcare.

## Figures and Tables

**Table 1:** Overview of pressure sensors and their performance parameters.

Sensor Type	Material	Structure	Pressure Range (kPa)	Sensitivity	Response Time (ms)	Refs.
Piezoresistive	MXene, ANF	Composite film	0–30	208.7 kPa^−1^	10	[12]
Piezoresistive	MXene, PU	Spiny microstructure	0–20	509.8 kPa^−1^	67.3	[17]
Capacitive	MWCNT, PDMS	Wrinkled dielectric layer	0.005–21	1.448 kPa^−1^	123	[7]
Iontronic capacitive	PVA, H_3_PO_4_	Hierarchical microstructured ionic film	0.00008–360	3302.9 kPa^−1^	9	[6]
Piezoelectric	PVDF	Multilayer row–column electrode array	80–750	7.7 mV·kPa^−1^	10	[26]
Triboelectric	Ecoflex, Ni textile	Pyramidal narrow-gap structure	0.8–81.6	~0.7 V at 1 N	—	[9]
Ionic–potentiometric	PVA electrolyte, PB/C, Ag/AgCl	Ionic layer/dual-electrode structure	0–625	205.5 mV/N (0–1 N)	71	[27]
Ionic–piezoionic	Crosslinked poly(ionic liquid)	All-solid-state film	0–80	~190 mV at 80 kPa	—	[28]
Electrochemical	Graphite/PDMS, Al, hydrogel	Porous sponge sandwich structure	0–110	10 kPa^−1^	83	[29]
Electrochemical	Mg, Cu, LiCl/PVA/CNT filter paper	High-power sandwich structure	0.6–100	0.738 kPa^−1^	—	[30]

Note: The quantitative values listed in this table are representative results from the cited literature. Since different studies employ different sensing mechanisms, output definitions, sensitivity units, device structures, and testing conditions, these values were not obtained under identical conditions and therefore should not be directly compared quantitatively.

**Table 2:** Common substrate materials and selection considerations for flexible pressure sensors.

Substrate	Preparation	Advantages	Limitations	Suitable Applications	Selection Notes
PDMS	Siloxane crosslinking and curing	Flexible, transparent, biocompatible, easy to microstructure	Poor breathability; viscoelastic hysteresis	Skin-mounted sensing, physiological monitoring, transparent tactile arrays	Improve breathability and adhesion for long-term use
PI	Polycondensation and thermal imidization	Heat-resistant, insulating, chemically stable	High modulus; limited stretchability	High-temperature and industrial pressure sensing	Unsuitable for large deformation
Ecoflex	Two-part silicone curing	Soft, highly stretchable, conformable	Creep; limited wear resistance	Skin sensing, soft robotics, physiological monitoring	Consider hysteresis and cyclic durability
PET	Polycondensation and melt extrusion	Transparent, low-cost, easy to pattern	Limited stretchability and heat resistance	Transparent tactile arrays, flexible electronics	Suitable mainly for bending deformation
PVA	PVAc hydrolysis and film/gel formation	Transparent, hydrophilic, biocompatible, easy to gel	Dehydration; poor environmental stability	Iontronic sensing, physiological monitoring	Encapsulation or moisture retention is often required
PAAm	Acrylamide crosslinking polymerization	Soft, highly hydrated, tunable	Dehydration, swelling, limited mechanical stability	Skin-mounted and low-pressure sensing	Requires anti-drying and anti-swelling treatment
PEN	Polycondensation and film formation	Heat-resistant, insulating, dimensionally stable	Relatively costly; limited stretchability	Elevated-temperature sensing, stable sensor arrays	Unsuitable for highly stretchable devices
TPU	Melt extrusion, hot pressing, or solution casting	Flexible, wear-resistant, fatigue-resistant	Hysteresis; temperature dependence	Long-term wearable sensing, motion monitoring	Suitable for repeated deformation
Textile/ nonwoven	Weaving, knitting, or fiber-web formation	Breathable, lightweight, comfortable	Limited uniformity and wash durability	Wearable sensing, plantar-pressure monitoring	Improve coating adhesion and washability

**Table 3:** Comparison of the performance, advantages, and limitations of flexible pressure sensors with different structural designs.

Structure	Materials	Sensitivity	Detection Range	Durability	Advantages	Limitations	Refs.
Serpentine	PI/Au/carbon–graphite composite	12.43 kPa^−1^	Up to 1 MPa	>12,000 cycles	Reduces strain interference	Complex fabrication	[18]
3D porous	Graphene foam [98]; PI fiber/CNT aerogel [99]	0.42 kPa^−1^ [98]	Up to 42 kPa [98]; 10 Pa–1.58 MPa [99]	10,000 cycles [98]	Wide range; high compressibility	Hysteresis; pore nonuniformity	[98,99]
Crack	MWCNT/PDMS composite	18.092 kPa^−1^	Up to 400 kPa	50,000 cycles	High sensitivity	Risk of crack propagation	[14]
Wrinkle	AgNWs/PDMS composite	0.947 kPa^−1^	Up to 50 kPa	-	Increased contact area	Difficult morphology control	[15]
Kirigami	Silver-coated PVDF film	0.025–0.056 V·N^−1^	Up to 1356 kPa	6000 cycles	Enhanced piezoelectric output	Complex cutting and assembly	[16]

Note: The quantitative values listed in this table are representative results from the cited literature. Since different studies employ different sensing mechanisms, output definitions, sensitivity units, device structures, and testing conditions, these values were not obtained under identical conditions and therefore should not be directly compared quantitatively.

## Data Availability

No new data were created or analyzed in this study. Data sharing is not applicable to this article.

## Abbreviations

The following abbreviations are used in this manuscript:

3D	Three-Dimensional
AI	Artificial Intelligence
AgNW	Silver Nanowire
ANF	Aramid Nanofiber
BP	Blood Pressure
CB	Carbon Black
CNN	Convolutional Neural Network
CNT	Carbon Nanotube
CVD	Cardiovascular Disease
DBP	Diastolic Blood Pressure
DIW	Deionized Water
EDL	Electric Double Layer
EMG	Electromyogram
E-jet	Electrohydrodynamic Jet
FCNT	Functionalized Carbon Nanotube
HMI	Human–Machine Interface
Hf-SiO_2_	Hafnium-doped Silicon Dioxide
LED	Light-Emitting Diode
LSTM	Long Short-Term Memory
LMMRE	Liquid Metal-filled Magnetorheological Elastomer
MAP	Mean Arterial Pressure
MAX	MAX Phase; M = transition metal, A = A-group element, X = carbon or nitrogen
MWCNT	Multi-Walled Carbon Nanotube
MXene	Two-Dimensional Transition-Metal Carbide, Nitride, or Carbonitride
PAAM	Polyacrylamide
PDA	Polydopamine
PDMS	Polydimethylsiloxane
PI	Polyimide
PS	Phenyl Silicone
PU	Polyurethane
PVA	Polyvinyl Alcohol
PVDF	Polyvinylidene Fluoride
PZT	Lead Zirconate Titanate
SBP	Systolic Blood Pressure
SEBS	Styrene-Ethylene/Butylene-Styrene
SEM	Scanning Electron Microscope/Scanning Electron Microscopy
SVM	Support Vector Machine
SWCNTs	Single-Walled Carbon Nanotubes
TENG	Triboelectric Nanogenerator
TPMF	Thermoplastic Polyurethane/Polydopamine/MXene Foam
TPU	Thermoplastic Polyurethane
WPBPS	Wearable Piezoelectric Blood Pressure Sensor
